# Radioiodine for thyroid cancer: sometimes, less is best

**DOI:** 10.1590/2359-3997000000164

**Published:** 2016-01-01

**Authors:** Rosalia do Prado Padovani

**Affiliations:** 1 Unidade de Endocrinologia e Serviço de Medicina Nuclear Faculdade de Ciências Médicas Santa Casa de São Paulo São Paulo SP Brasil Unidade de Endocrinologia e Serviço de Medicina Nuclear, Faculdade de Ciências Médicas da Santa Casa de São Paulo (FCMSCSP), São Paulo, SP, Brasil

This issue of *Archives of Endocrinology and Metabolism* contemplates two important articles that discuss controversies related to the indication of radioactive iodine (RAI) therapy for patients with differentiated thyroid carcinoma (DTC), highlighting the need of future studies in this area, which still has many points to be questioned and discussed.

The first article, “Is ^131^I ablation necessary for patients with low-risk papillary thyroid carcinoma and slightly elevated stimulated thyroglobulin after thyroidectomy? (1) presents a very controversial and interesting issue. Emphasizing the discussion on the real benefit of RAI ablation for some DTC patients, the study assessed the risk of disease recurrence in 53 low risk patients, who, after surgery, remained with slightly elevated stimulated Tg. The time of follow-up ranged from 36 to 96 months. The results demonstrated that the great majority of the patients had a good evolution during the follow-up even without receiving RAI ablation.

In fact, some previous studies have shown that low-risk patients who develop postoperative stimulated thyroglobulin (Tg) ≤ 1 ng/mL in the absence of anti-Tg antibodies (TgAb) and negative ultrasonography (US) postoperative do not benefit from RAI ablation because recurrence seldom occurs in this group (2-4). Even the 2015 ATA guideline (5) and the 2013 DTC Brazilian Consensus (6) recommend the same guidance. However, only two previous studies evaluated the follow-up of low risk patients who were not submitted to ablation because of low postoperative Tg (4,7). This remark emphasizes the importance of this prospective study by Rosario and Mourão (1), which also showed a low recurrence rate (approximately 2%) in a large number of low risk patients who, following total thyroidectomy without central neck dissection, remained with slightly elevated stimulated Tg and were not submitted do RAI ablation.

Another important contribution of this study was the confirmation of the stimulated Tg values cut off as a parameter to spare low-risk patients from ablation. The cut off (stimulated Tg > 1 ng/mL, but ≤ 5 ng/mL after L-T4 withdrawal or ≤ 2 ng/mL after rhTSH) was the same used in two previous studies (4,7) and was again considered a good marker to distinguish normal residual thyroid tissue from persistent thyroid neoplastic disease.

Even considering that, in the near future, current functional assays with higher sensitivity of Tg (sensitivity < 0.1 ng/mL) will likely replace the use of stimulated Tg, most services still use, as a routine, Tg stimulation to confirm any evidence of disease. These new assays tend to obviate the need for rhTSH stimulation in low risk patients with a Tg on levothyroxine (LT4) treatment below 0.1-0.2 ng/mL (5).

The periodic Tg measurements on thyroid hormone therapy are also very important in the follow-up of patients and were also emphasized by this study. While specific cutoff levels of Tg that optimally distinguish normal residual thyroid tissue from persistent thyroid cancer are unknown, the observation of rising Tg values over time can also be used as a marker of suspicious growing thyroid tissue or cancer. For patients who present elevated Tg values over time, especially those with increasing Tg behavior and negative cervical US, remnant ablation or adjuvant therapy with ^131^I may be considered. There is no evidence in these low risk patients that a delayed treatment may adversely affect the outcome or decrease the chance of cure (5).

Other information highlighted in the study was the importance of continuous follow-up of patients with cervical US. Although prophylactic dissection of the cervical lymph nodes was not performed in the patients of the study, lymph node metastases could be well diagnosed by cervical US, confirming that this assessment is considered an important and essential surveillance tool in the follow-up of papillary DTC patients. It is also important to remember that serum Tg measurements obtained during thyroid hormone suppression of TSH, and, less commonly, following TSH stimulation, may fail to identify patients with small amounts of residual tumor (8). Even in these cases, with serum undetectable Tg, the cervical US can help to recognize these minimal amounts of residual disease and exclude the possibility of neoplastic disease (9).

Another point to be raised in this study is the inclusion of intermediate risk patients [26 patients presented tumors > 4 cm or minimal extra thyroid invasion (pT3, cN0pNx, M0)] in the selected group of patients classified as low risk (1,9). By including these patients, the author also evaluated the recurrence rate of this intermediate risk group that remained with slightly elevated stimulated Tg after surgery. And, not surprisingly, the only patient who had lymph node recurrence came from this group, even though with a favorable evolution. This observation suggests that some patients initially considered as being intermediate risk who develop slightly elevated Tg can also have an excellent outcome, indicating the possibility that this group of patients may be just followed without RAI ablation. This is certainly a controversial topic that requires further investigation to be well-defined.

In conclusion, the results obtained from this study suggests that low-risk and some intermediate risk patients who present stimulated Tg ≤ 5 ng/mL after L-T4 withdrawal or ≤ 2 ng/mL after rh TSH in the absence of TgAb and combined with negative neck US after thyroidectomy can be initially followed without RAI ablation. Nevertheless, even for these patients, it appears reasonable to consider a periodic follow-up with assessment of Tg on LT4 treatment, TgAb and US as a strategy for surveillance.

The second study, “Is radioactive iodine^-131^ treatment related to the occurrence of non-synchronous second primary malignancy in patients with differentiated thyroid cancer? (10), also presented a very controversial and interesting subject that challenges physicians treating DTC patients. The authors evaluate a large number of patients (n = 413) with histopathological diagnosis of thyroid cancer treated between January 1^st^ of 1979 and December 31 of 2009. From the entire group, 413 had all the criteria for inclusion and were followed for at least 3 years (mean follow-up period was 11.0 ± 7.5 years). The results showed an incidence of 4.1% non-synchronous second primary malignancy (NSSPM) related to the occurrence of solid malignancies. Nevertheless, no statistically significant association between RAI treatment or cumulative ^131^I activity and NSSPM occurrence was observed. An interesting observation of the study was that, probably, a tendency of premature NSSPM occurrence among patients treated with RAI exists. The author also concluded that patients with more advanced age at DTC diagnosis were under greater risk of NSSPM development (10).

This study is considered relevant because DTC incidence has been rising and its overall prognosis after treatment is favorable with a low mortality rate (11). Therefore, the great majority of patients with DTC is candidate to a long-term survival, which can influence the potential for development of secondary malignancies (12).

Although RAI has been used in medicine for over half a century (13), evidences about possible carcinogenic effects after its administration for therapeutic purposes are still a matter of debate. Several studies have described an increased association of second primary malignancies following diagnosis of thyroid cancer but, still, there is no confirmation about the association with RAI therapy (14,15). The difficulty in reaching a conclusion probably is related to the long latency period for the clinical appearance of radiation induced tumors, especially solid tumors (13). Therefore, it would be necessary to follow a great number of patients for many decades to be able to show, conclusively, whether an excess of second tumors occurs in patients treated with ^131^I when compared to a control population. This is especially difficult when dealing with relative low prevalent tumors such as thyroid carcinoma (16). This study by Souza and cols. (10) encompassed a considerable time of follow-up (11.0 ± 7.5 years since diagnosis), but insufficient to evaluate second primary malignancy tumors occurrence in DTC patients.

Another bias of this kind of study concerns the heterogeneity of data obtained over long periods of time due to the improvement of diagnostic and therapeutic tools. Moreover, the exposure to other carcinogenic factors makes the assessment even more difficult.

Another point to be considered concerns how differences in clinical, pathologic and treatment characteristics alter the risk profiles for developing second primary cancers. In this study, the author aimed to evaluate variables other than ^131^I that could be associated with NSSPM, such as age, gender and DTC histological subtype. Age at DTC diagnosis was the only independent factor significantly associated with NSSPM. Nevertheless, the relative small number of patients (just 17 patients presented NSSPM) could influence the results. Another important limitation of the study was that no active investigation for secondary malignances was performed, what raises the possibility of silent tumors not being considered in the analyses.

In conclusion, this study suggests that the ^131^I may play a role in anticipating NSSPM occurrence, what is considered an important negative effect of RAI therapy. More studies are needed to confirm or disprove this theory and investigate molecular-genetic and environmental factors that may aid in the identification of specific groups at higher risk of developing a second primary cancer. Meanwhile, it is interesting that all patients with DTC undergo specific screening for common second primary cancers as leukemia, breast and prostate for example.
